# Interleukin 4 Affects Epilepsy by Regulating Glial Cells: Potential and Possible Mechanism

**DOI:** 10.3389/fnmol.2020.554547

**Published:** 2020-09-04

**Authors:** Lu Chen, Lin Zhu, Di Lu, Zhe Wu, Yanbing Han, Puying Xu, Lvhua Chang, Qian Wu

**Affiliations:** ^1^Department of Neurology, First Affiliated Hospital, Kunming Medical University, Kunming, China; ^2^Biomedicine Engineering Research Centre, Kunming Medical University, Kunming, China; ^3^Department of Psychology, The First People’s Hospital of Yunnan Province, Kunming, China

**Keywords:** interleukin 4, epilepsy, microglia, astrocytes, cognition

## Abstract

Epilepsy is a chronic brain dysfunction induced by an abnormal neuronal discharge that is caused by complicated psychopathologies. Recently, accumulating studies have revealed a close relationship between inflammation and epilepsy. Specifically, microglia and astrocytes are important inflammatory cells in the central nervous system (CNS) that have been proven to be related to the pathogenesis and development of epilepsy. Additionally, interleukin 4 (IL-4) is an anti-inflammatory factor that can regulate microglia and astrocytes in many aspects. This review article focuses on the regulatory role of IL-4 in the pathological changes of glial cells related to epilepsy. We additionally propose that IL-4 may play a protective role in epileptogenesis and suggest that IL-4 may be a novel therapeutic target for the treatment of epilepsy.

## Introduction

Epilepsy is a chronic neurological disease characterized by recurrent seizures. There are 65 million people worldwide that currently suffer from epilepsy, 80% of whom live in developing countries. Furthermore, recurrent seizures and related cognitive impairments lead to a significant social and economic burden (Beghi, 2020). At present, it is thought that disorders in synaptic structure and neuronal excitability might be involved in epileptogenesis (González et al., 2019). Moreover, the inflammatory and immune responses in the early stage of human brain development have been proven to result in several neuropsychiatric diseases and dysfunctions such as autism, schizophrenia, cerebral palsy, epilepsy, cognitive disorders, depression, et cetera (Mahfoz and Shahzad, 2019). Accumulating evidence has recently verified that neurons and endothelial cells of the blood-brain barrier (BBB), activated microglia and astrocytes contribute to neuroinflammation in animal models with epilepsy. Furthermore, the inflammatory cascade and its related processes play important roles in epileptogenesis and epilepsy-related cognitive impairment (Vezzani et al., 2019). For instance, after epileptic injury [such as neurotrauma, stroke, central nervous system (CNS) infection], status epilepticus (SE), febrile seizures (FS) and recurrent seizures, glial cells are activated and subsequently followed by the release of large amounts of cytokines (CKs), chemokines, and the activation of downstream reactions that eventually worsen the onset of primary seizures or cause secondary seizures (Vezzani et al., 2013). Also, several pro-inflammatory CKs, such as interleukin 1β (IL-1β) and tumor necrosis factor-α (TNF-α), are involved in decreasing the seizure threshold and leading to epileptogenesis (Iori et al., 2016). Interleukin 4 (IL-4) is an anti-inflammatory cytokine that has been proven to reduce the activation of various immunocompetent cells such as macrophages, monocytes, and neutrophils by inhibiting the production of pro-inflammatory CKs (IL-1, TNF, etc.; Standiford et al., 1990; Te Velde et al., 1990; Wertheim et al., 1993). In the CNS, Park et al. (2015) also found that IL-4 could inhibit IL-1β-induced depression-like behavior, modulate the metabolism of corticosterone (CORT), prostaglandin E2 (PGE2), serotonin (5-HT), norepinephrine (NE) and other hormones and neurotransmitters in a rat disease model.

Currently, many studies are focusing on the relationship between IL-4 and epilepsy. In a study of 82 Iranian FS children aged 6 months to 6 years old, Zare-Shahabadi et al. (2015) evaluated allele and genotype frequencies of three single-nucleotide polymorphisms of the IL-4 gene. This work found that, compared to controls, the level of IL4-590/C allele and TCC haplotype was higher and the frequencies of GCC, TTT, TTC haplotypes, and that the IL-4 (-590) TC, IL-4 (-33) TC genotypes were lower in epilepsy patients. This study illustrated that the IL-4 gene changes in FS patients and may make individuals more susceptible to the disease (Zare-Shahabadi et al., 2015). However, Tsai et al. (2002) detected IL-4 intron three gene polymorphism in 51 FS and 43 epileptic children from Taiwan and found that there was no significant difference between patients with healthy children. Therefore, they concluded that the association of IL-4 polymorphisms with FS and epilepsy of children does not exist (Tsai et al., 2002). Both experiments use polymerase chain reaction monitoring, which may cause different results due to the patient’s ethnicity and different detection sites, alleles, and genotypes. Moreover, Ha et al. (2018) tested IL-4 levels in 50 FS patients within 1 h of a seizure and found that IL-4 levels were higher in those patients who had fever without seizures. Similarly, in the serum of 22 out of 100 patients with epilepsy, IL-4 was significantly increased within 24 h of seizures. During the seizure-free period, the level of IL-4 could be reduced from 18.8 to 0% of the pre-seizure period (Table 1). These results all point to the concept that the increase of anti-inflammatory cytokine IL-4 may be a natural defense mechanism as the body responds to injury. On the other hand, due to the multimodal effect of CKs, IL-4 can play an anti-inflammatory or pro-inflammatory function in different environments (Sinha et al., 2008). For example, Li et al. (2017) found that in mice, spontaneous recurrent seizures induced by the intraperitoneal injection of pilocarpine, which up-regulates IL-4, can rescue microglial phenotypes, reduce the frequency, duration and severity of spontaneous recurrent seizures, and improve cognitive dysfunction. Thus, IL-4 likely plays an important role in epileptogenesis and the physiopathology of epilepsy. The current review aims to elaborate on the possible role of IL-4 in epileptogenesis, epileptic development, and epilepsy-related cognitive impairment.

**Table 1:** IL-4, glial cells, and epilepsy.

Epilepsy	Interleukin 4 (IL-4) related mechanism	Glial cell targets	Epilepsy related outcome	Research model
FS	IL-4 polymorphisms	-	-	Human
Partial seizure	IL-4 increase	-	-	Human
TLE	IL-13 increase	M2 polarization of	-	KA-induced mice
		Microglia/macrophages;	-	
		M1 inhibition	Reduced frequency, duration, and severity of spontaneous epilepsy	Lithium-pilocarpine induced rats
SE	IL-4 increase;	M1, M2	Reduce frequency, duration,	Human
	IL-1β increase	M1 inhibition	and severity of spontaneous	KA-induced mice
			Recurrent seizures; improve	Pilocarpine-induced mice
			Cognitive dysfunction	
	HMGB1-mediated pathway	Astrogliosis, microgliosis		Pilocarpine-induced Wistar rats
Intractable epilepsy	IL-4 increase	Astrocytic hypertrophy and proliferation	Inhibited the mitogenic effect of TNF on astrocytes	Non-neoplastic human astrocytes

## Il-4

### Overview

IL-4 is a pleiotropic cytokine mainly produced by activated T lymphocytes, especially Th2 cells, mast cells, and basophils. Isakson et al. (1982) found that a cytokine can promote B cell proliferation, which was successfully cloned in 1986 and eventually named IL-4 (Isakson et al., 1982). Although the IL-4 cDNA sequence in human beings contains 153 amino acid residues, it can only produce secreted IL-4 proteins containing 129 amino acid residues. The spherical hydrophobic core of secreted IL-4 contains three disulfide bonds and four α-helices (Powers et al., 1992). After the addition of N-chain oligosaccharides, this core produces different molecular weights that include 15, 18, and 19 kDa. Essentially, secreted IL-4 mainly functions as follows: (1) driving Th2 cell differentiation to produce more IL-4, and then promoting the release of other Th2 anti-inflammatory CKs such as IL-5 and IL-13; (2) inhibiting the production of pro-inflammatory CKs such as TNF-α, interferon γ (IFN-γ), and IL-17; and (3) promoting B cell proliferation and differentiation and up-regulating the expression of major histocompatibility complex II (MHC II) molecules, IL-4R and CD23 on B cells (Lu et al., 2015).

### IL4-Related Signaling Pathways

**Figure 1:**
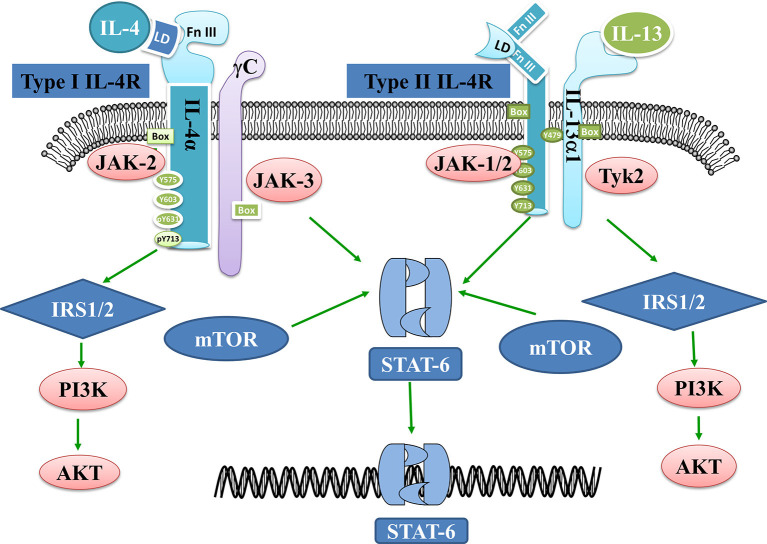
Interleukin 4 (IL-4)- and IL-13-related signaling pathways. IL-4 binds to Type I receptors and then activates the IRS1/2-P13K-AKT and STAT6 pathways. mTOR is a prime synergistic protein that helps IL-4 to maximally activate STAT6. IL-13 on the other hand binds to Type II receptors and activates JAK1/2 and Tykine2 (tyrosinekinase2), which in turn activates the IRS1/2-P13K-AKT or STAT6 pathways.

There are two types of IL4 receptors, Type I and Type II. Type I receptors are a kind of heterodimer receptor that was formed by IL-4 receptor α (IL4Rα) and common r chain (γc) chains and are mainly expressed on the surface of hematopoietic cells. Type II receptors contain the IL4Rα and IL-13 receptor α1 (IL-13Rα1) and are expressed on the surface of non-hematopoietic cells (Sequeida et al., 2020). The binding of IL-4 to its receptor leads to the activation of the Janus kinase (JAK) family and the phosphorylation of IL4Rα. Three signaling pathways are related to the activation of the JAK family: (1) insulin receptor substrate protein (IRS)/Phosphoinositide-3 kinase (PI3K)/Protein kinase B (AKT) pathway. Activated JAK3 can stimulate IRS tyrosine phosphorylation and provide binding sites to those signaling molecules which contain the SH2 domain, such as P85. Then, the combination of P85 and IRS can activate PI3K and its downstream signaling molecules such as AKT, and finally affect cell proliferation and differentiation. (2) Signal transduction and activation of the transcription factor (STAT6) pathway. STAT6 forms a homomeric dimer by JAKs phosphorylation which is then transported to the nucleus initiates IL4/IL13 gene transcription and regulates the expression of genes such as CD23 (Keegan et al., 2018). (3) Mammalian target of rapamycin (mTOR) signaling pathway. mTOR is a critically synergistic protein that aids IL-4 in activating STAT6 to the greatest extent and ultimately regulates the differentiation of Th2 cells (Delgoffe et al., 2009). In short, IL-4 regulates the growth and development of T cells through a variety of molecular signal pathways, but its specific mechanism remains unknown (Figure 1).

Besides, IL-4 can also abnormally activate STAT3 in glioblastoma (GBM) cells, which are related to the expression of IL-13Rα2 (Rahaman et al., 2005). In most cases, STAT3 mediates pro-IL-6 and anti-IL-10 signals (Pfitzner et al., 2004). It was reported that pilocarpine-induced SE can activate the JAK/STAT signaling pathway, especially STAT3 (Xu et al., 2011). Similarly, Zhang et al. (2019) found that STAT3 was overexpressed in 169 children suffering from epilepsy, suggesting that it is associated with the risk of epilepsy and drug resistance to epilepsy (Li et al., 2020). By inhibiting STAT3 gene transcription, the frequency of seizures can be reduced (Grabenstatter et al., 2014).

### IL-4, IL-13, and Epileptogenesis

IL-4 and IL-13 are related CKs that may activate similar downstream signaling pathways and exert molecular regulatory effects (Athari, 2019). IL-13Rα1 can specifically bind to IL-13 through its critical binding unit D1 domain. This can lead IL-13 to play an important role in the pathogenesis of bronchial asthma by acting on epithelial cells and fibroblasts (Ito et al., 2009). Moreover, Umeshita-Suyama et al. found that IL-4 and IL-13 induced STAT3 activation in B cells with high expression of IL-13Rα1 (Umeshita-Suyama et al., 2000). IL-13Rα2 is another receptor of IL-13; Andrews et al. (2013) used surface plasmon resonance (SPR) analysis to show that IL-13Rα2 does not bind IL-4, nor does it affect the binding of IL-4 to IL-4Rα. These authors also used EBAS-2B cell lines (human bronchial epithelium) to prove that IL-13Rα2 overexpression weakened IL-4 and IL-13-mediated STAT6 phosphorylation. Furthermore, IL-13Rα2 without cytoplasmic domain continued to weaken the IL-13-mediated signaling pathway but did not affect the IL-4 mediated STAT6 signaling pathway (Andrews et al., 2013).

Studies have shown that IL-13 also may be involved in the regulation of the inflammatory process of the CNS, however, whether this regulatory role is protective or disruptive is controversial. In a study on experimental allergic encephalomyelitis (EAE) induced by myelin oligodendrocyte glycoprotein (MOGp), Barik et al. (2017) found that the ability of Th17 cells from IL-13Rα1–deficient (13R^−/−^) mice to transform into Th1 cells was reduced, and the sensitivity to Treg inhibition was also reduced. HR (13R^−/−^) mice were more susceptible to EAE and developed early-onset and more severe disease. These observations indicated that IL-13 can control immune-mediated CNS inflammation (Barik et al., 2017). Le Blon et al. (2016) transplanted mesenchymal stem cells (MSCs)/IL-13 into a Cuprizone (CPZ) mouse model and found that IL-13 released by grafted MSC was able to trigger the alternate activation of macrophages and microglia related to MSC transplantation. IL-13 was also found to reduce the inflammation and demyelination induced by CPZ through its direct effect or the combined effect with the alternate activation of macrophages/microglia, indicating that IL-13 has protective effects on CPZ-induced neuroinflammation (Le Blon et al., 2016). Some studies have found that the CPZ mouse model showed tonic-clonic seizures, intermittent ictal spikes, and frequent spike discharge. Accordingly, CPZ models have also been used to study pathology and/or therapy for epilepsy (Praet et al., 2014). However, after transplanting MSCs/IL-13 into mice hippocampi and then inducing epilepsy by Kainic acid (KA) 1 week later, Ali et al. (2017) found that transplantation had no significant influence on the duration, frequency, and onset of seizures, or their electroencephalographic (EEG) dynamics. Hence, they claimed that the injection of MSCs/IL-13 into the hippocampus might not have a direct protective effect on SE or chronic epilepsy. Nevertheless, as an indirect mechanism, MSCs/IL-13 was found to optimize the hippocampal niche, outside of the lesion site, by inducing M2 polarization of microglia/macrophages (Table 1, Ali et al., 2017). Several aspects, such as the difference in the transplanted cell line, transplantation methods, grafting sites and EEG monitoring time might lead to different or even contrary results. On the one hand, MSC grafts and epilepsy may result in large astrocyte scars which can physically obstruct IL-13 from reaching the hippocampus. On the other hand, neuroinflammation associated with MSC transplantation may counteract the protective effect of IL-13 on inflammation. Therefore, whether IL-13 has a protective effect on the pathophysiology of epilepsy is still controversial, and more powerful evidence is needed for verification.

## Il-4, Glial Cells, and Epilepsy

### IL-4, Epilepsy, and Microglial Phenotypic Transformation

**Figure 2:**
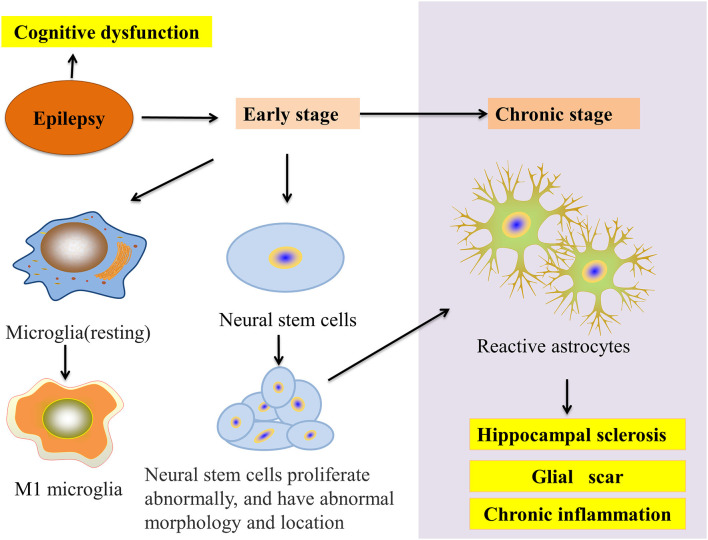
Glial cells and epilepsy pathology. In the early stage of epilepsy, microglial cells transform into the M1 phenotype and neural stem cell (NSC) disorders appear in proliferation, migration, and integration. In chronic epilepsy, neurogenesis gradually decreases to a halt. NSCs start transforming into reactive astrocytes and finally accelerate HS. Moreover, these processes result in the epilepsy-induced inhibition of autophagy and cognitive impairment.

Microglia and astrocytes are the main inflammatory cells in the nervous system. Glia-mediated inflammation induced by various brain insults can promote seizures and epileptogenesis (Figure 2), especially when the inflammation is difficult to control (Eyo et al., 2017). Microglia are highly adaptable glial cells that, during development, recognize and phagocytose apoptotic neurons, prune synapses, modulate the differentiation and migration of neural precursor cells, regulate neurogenesis, and improve neuron survival. In the mature brain, microglia play an important role in general cognitive function, learning and memory, neuroplasticity, and synaptic plasticity (Hammond et al., 2018). Moreover, microglia can regulate the release of anti-inflammatory and pro-inflammatory factors. During brain and niche damage, microglia can be activated through morphological changes or molecular modifications, which can activate a protective response to inflammation in the CNS (Tay et al., 2017). Interestingly, microglia can exhibit different activation subtypes—M1, M2a, M2b, M2c—according to environmental changes and exogenous stimuli. The M1 phenotype is mainly induced by lipopolysaccharides (LPS) and IFN-γ; the M2a phenotype by IL-4 and IL-13; the M2b phenotype by the immune complex toll-like receptor and IL-1R agonist; the M2c phenotype by IL-10 and glucocorticoids (Franco and Fernández-Suárez, 2015).

RNA sequencing analysis showed that microglial phenotypes varied according to their response function in patients who suffered from mesial temporal lobe epilepsy with hippocampal sclerosis (MTLE-HS; Morin-Brureau et al., 2018). For example, in CA1/CA3 after epilepsy, microglial cells could reduce neuron density by regulating MHCII overexpression and their shapes. Furthermore, increased adenosine diphosphate (ADP) can induce microglial migrative behavior by expressing purinergic receptor 1 (P2Y1; De Simone et al., 2010), enhance tissue repair, promote vascular reconstruction and strengthen the BBB by expressing IL-10 and its related transcription factors. In different epilepsy models, microglia phenotypes undergo similar transformations. In a study of the Cstb^−/−^ mouse, an animal model for progressive myoclonus epilepsy of Unverricht–Lundborg type (EPM1), Okuneva et al. (2015) found that the proportion of pro-inflammatory M1 and anti-inflammatory M2 microglia was higher than that of the control group. Additionally, in pre-symptomatic Cstb^−/−^ mice, M1/M2 polarization is skewed towards the M2 type at postnatal day 14 (P14). At P30, however, this ratio reversed to skew towards M1, which is a time point related to the onset of myoclonus. This phenomenon indicates that M2 microglia attempt to support neurons that may be dysfunctional and prevent further damage, however, may fail to maintain this function (Okuneva et al., 2015). Benson et al. (2015) compared microglial polarization in pilocarpine-induced SE and kainic acid-induced SE. In the acute phase, both M1 and M2 marker expression was increased in the pilocarpine model, while M1 in the KA model increased. This difference may be due to the more frequent seizures in the KA model. Consequently, acute M1 upregulation post-SE can play an important role in epileptogenesis (Benson et al., 2015). In a rat lithium-pilocarpine model of TLE, Wang et al. (2015) found that M1 microglial cells maintained a high level at 7 and 14 days after SE. Subsequently, they proved that the second generation of tetracycline-minocycline can effectively inhibit M1 microglia activation, mitigate SE induced brain inflammation, and reduce the frequency, duration, and severity of spontaneous epilepsy (Wang et al., 2015). Hence, correcting phenotypic deviations when the microglia phenotype balance changes will help improve the prognosis of epilepsy.

The interleukins IL-10, IL-13, and IL-4 are essential modulators of neuroinflammation by promoting the polarization of M2-like microglia (Michels et al., 2014). In an epilepsy mouse model, Li et al. (2017) found that M1 and M1-associated cytokine IL-1β rapidly increased to the peak value 1 day after SE, and fell to normal levels in 3 weeks. On the other hand, IL-4 was unchanged in the early epileptic stages and gradually increased over the following 2 weeks. However, the percentage of M2 exhibited an immediate drop on the first day after SE and then gradually increased at 3 weeks after SE. Nevertheless, based on the intraperitoneal injection of IL-4 (100 ng/mouse) 5 h before and 4 days after pilocarpine-induced SE, Li et al. (2017) found that IL-4 inhibited the early increase of M1. By contrast, except for the first day after IL-4 injection, M2 did not show a substantial sustained increase after SE. Therefore, Li et al. (2017) considered the reduced damage and inflammation due to early inhibition of M1 which may inhibit the subsequent increase in M2. This study first indicated that IL-4 can affect the inflammatory process of epilepsy by regulating microglial phenotypes (Table 1, Li et al., 2017).

Additionally, in a stroke model, early intracerebral injection of IL-4 was able to inhibit M1 activation while enhancing M2 microglial activation and promoting neuro-functional recovery (Yang et al., 2016). Knock-out STAT6 mice led to the down-regulation of IL-4 and STAT6/Arg1 (arginase 1), which stimulates the transformation of microglia and macrophages into pro-inflammatory phenotypes and result in the reduction of dead/dying neuron clearance in the lesion area, augmentation of brain inflammation, increase of neuronal death, and eventually poor long-term prognoses (Cai et al., 2019). In an Alzheimer’s diseases (AD) mouse model, IL-4 did induce a robust M2a phenotype (Latta et al., 2015), and rather induced autophagy vacuole formation and microglial autophagy flux generation, increased uptake and degradation of Amyloid-β (Aβ), inhibited the Aβ deposition induced autophagy flux blockade, and returned autophagy flux to a normal level (Tang et al., 2019). Based on the above results, IL-4 can likely regulate the microglia to the anti-inflammatory M2 phenotype, enhance the phagocytosis of apoptotic neurons, and play a protective role in many neurological diseases such as epilepsy, stroke, and AD. Additionally, epidemiological studies have shown that the risk of epilepsy among brain-infected survivors is 7–9% in developed countries and is much higher in developing countries (Ramantani and Holthausen, 2017). Neuronal excitability secondary to pro-inflammatory signals caused by CNS infection is an important mechanism for epileptogenesis (Singhi, 2011). Stroke is responsible for approximately 10% of all seizures and 55% of new seizures in the elderly. Early epilepsy after stroke is caused by local metabolic disorders, without changing the neural network and late epilepsy by acquired epilepsy susceptibility (Feyissa et al., 2019). Furthermore, in patients over 65 years of age, neurodegenerative diseases account for approximately 10% of all new seizures, recurrent cases of which also aggravate the decline in cognitive function (Friedman et al., 2012). The accumulation of Aβ peptides in the brain of AD patients can cause synaptic degeneration and remodeling of neuronal circuits, leading to neuronal hyperexcitability. As a result, epilepsy is highly correlated with neurological diseases such as infections, stroke, and AD (Garg et al., 2018). Nevertheless, the specific mechanism of IL-4 in epileptogenesis needs to be further studied.

### IL-4, Epilepsy, and Astrocytes

Astrocytes account for about one-third of brain cells and can provide structural, metabolic, and homeostatic support for neurons (Shigetomi et al., 2019). After infection, trauma, ischemia, and neurodegenerative disease, astrocytes are activated, and in severe cases, reactive astrocytes progressively enlarge, proliferate, and form marked scars (Sofroniew, 2009).

*In vivo* and *in vitro* experiments investigating epilepsy have revealed that reactive astrocyte proliferation and glial scar formation are common pathological changes. In a KA-induced mouse model of medial temporal lobe epilepsy (MTLE), Muro-García et al. found that at 1 week after epilepsy, neural stem cells (NSCs) began transforming into reactive NSCs that would gradually inhibit neurogenesis and transform into reactive astrocytes. Even more, at 6 weeks post-seizure, hippocampal neurogenesis completely stopped, and reactive astrocytes induced hippocampal sclerosis (HS) and chronic inflammation. Moreover, drug-resistant MTLE patients were found to have inhibited neurogenesis, reactive astrocyte proliferation, and HS (Muro-García et al., 2019). Interestingly, in both mitochondrial epilepsy (a type of epilepsy that is extremely difficult to treat and has a poor prognosis due to mutations in mitochondrial DNA) and Rasmussen’s encephalitis (RE; a type of epilepsy with chronic brain inflammation), patients experienced astrocyte apoptosis and loss (Bauer et al., 2007; Chan et al., 2019). Above all, reactive astrocytes play an important role in epileptogenesis and epileptic development. Based on these results, intervention in reactive astrocytes may become a new target for epilepsy treatment.

Early in 1993, in an adult human glial cell line-derived from epilepsy white matter, Estes et al. (1993) confirmed that IL-4 down-regulated the DNA synthesis and proliferation of astrocytes and inhibited the mitogenic effect of TNF on astrocytes by anti-IL-4 antibody. Moreover, in an AD model, IL4/STAT6 signaling hurt astrocyte survival *in vivo* (Mashkaryan et al., 2020), and *in vitro*, IL-4 rescued the impairment of proliferation and neurogenic ability of primary human cortical astrocytes by Aβ42-induced *via* IL-4/STAT6 pathway (Papadimitriou et al., 2018). On the other hand, Brodie et al. (1998) found that astrocytes express IL-4R *in vivo* but do not secrete IL-4. Furthermore, after being treated with IL-4, the activation of astrocytes was inhibited, NO, and iNOS content caused by LPS stimulation was decreased, and TNF-α secretion was reduced. These results indicate that IL-4 may act as an immunosuppressive factor in the CNS during inflammation (Brodie et al., 1998). Also, high mobility group box-1 protein (HMGB-1), an important damage-associated molecular pattern (DAMP), has been shown to induce dendrite loss and neurodegeneration *via* nuclear factor-κB (NF-κB) signaling activation. The release of NF-κB in human patients and models of epileptic seizures are often accompanied by reactive gliosis and neurodegeneration. In a model of pilocarpine-induced SE Wistar rats, Rosciszewski et al. (2019) found that blocking the HMGB1-mediated signaling pathway was beneficial to reduce reactive astrogliosis, microgliosis and neurodegenerative changes after SE. Furthermore, in glial cultures obtained from cerebral cortices of C57BL/6 mice treated with different concentrations of HMGB1, Yao et al. (2019) elucidated that IL4 could activate PPARγ *via* the STAT6 singling pathway and inhibit NF-kB activation, significantly reducing the formation of the HMGB1-mediated NOD-like receptor with pyrin domain containing-3 (NLRP3) inflammasome complex in astrocytes (Table 1). Additionally, Garg et al. (2009) found that IFN-γ protected the ability of murine astrocytes to clear extracellular glutamate *in vitro* and undergo oxidative stress and that IL-4 has no effect at any concentration that was tested (10–100 ng/ml). When IL-4 and IFN-γ were co-administered, IL-4 reduced the clearance of glutamate by IFN-γ. However, IL-4 avoided the harmful effects of excessively strong IFN-γ by increasing the neuroprotective thiol and lactic acid secretion and inhibiting the release of Th1 CKs (Garg et al., 2009). In summary, IL-4 can regulate reactive astrocytes and play a protective role against neuroinflammation through the IL-4/STAT6 signaling pathway. However, whether IL-4 has protective effects on reactive astrocyte hyperplasia and inflammation caused by epilepsy needs further verification.

Oligodendrocytes are myeloid cells in the CNS, which must undergo a complex and precise process of proliferation, migration, differentiation, and myelination, and finally form an insulating sheath (Bradl and Lassmann, 2010). In 30 patients who underwent surgical resection for intractable focal epilepsy, Sakuma et al. (2014) found that compared with control cases, the number of oligodendroglia-like cells (OLCs) in refractory focal epilepsy specimens of children increased, OLCs increased in the gray matter and the junction of gray/white matter to white matter. Zhang et al. (2019) further confirmed that IL-4 has a direct beneficial effect on the differentiation of oligodendrocytes through the PPARγ axis, however, at present, the effect of IL-4 on the increase of post-epileptic OLCs lacks relevant evidence.

### The IL4/IL4R Axis in the Treatment of Glioma and Glioma-Related Epilepsy

Gliomas are the most common primary CNS brain tumors and include astrocytomas, oligodendrogliomas, and ependymomas that originate in astroglial cells, oligodendrocyte cells, ependymal cells or cancer stem cells (Zhu et al., 2012). Increasing evidence suggests that the growth of gliomas stimulate seizures, while seizure activity may also contribute to tumor growth (Yang et al., 2016). Symptomatic seizure activity secondary to gliomas is referred to as glioma-associated epilepsy (GRE). The epileptogenesis of GRE involves multiple factors, including tumor location, degree of differentiation, tumor microenvironment, and specific genetic changes (Liang et al., 2019). In 65–90% of low-grade gliomas (LGG), epilepsy is the most common first symptom. Furthermore, seizure control is often the most important predictor of quality-of-life in patients with recurrent LGG (Dunn-Pirio et al., 2018). Compared to standard antiepileptic medications, effectively inhibiting the development of gliomas is more conducive to control the onset of glioma-related seizures (Samudra et al., 2019). Moreover, Zhu et al. (2012) indicated that CKs play a critical role in glioma diagnosis, prognosis, and therapy.

From 100 histologically confirmed adult Iraq patients with glioma blood samples, Shamran et al. (2014) suggested that the C allele of the SNP S503P in the IL-4R and the T allele of the SNP C-33T in the IL-4 gene may have a protective role against glioma development. Compared with normal brain tissues, IL-4R is overexpressed in GBM; based on this phenomenon, Rand et al. (2000) developed a cytotoxin targeting IL-4R and cpIL4-PE, which can mediate extensive necrosis of gliomas without obvious toxicity to healthy adjacent brain tissue. Joshi et al. (2001) demonstrated that human brain tumors *in situ* also overexpressed IL-4R and that most GBM primary cell cultures were found to be highly sensitive to cpIL4-PE, but not to normal astrocytes or neuronal cell lines. Hence, these results confirmed that the differential expression of IL-4R may offer an attractive target of IL-4 cytotoxin for brain tumor therapy (Joshi et al., 2001). In recent years, tumor drugs have targeted AP1, a new glioma affinity peptide that specifically binds to IL-4R and exhibits the highest therapeutic effect on glioma (Sun et al., 2017). Moreover, early in 2000, Liu et al. (2000) revealed that IL-4α receptors in non-neoplastic astrocytes derived from human brain specimens of patients with epilepsy were expressed similar to malignant astrocytoma (Table 1). Therefore, IL-4R may also be a new target for glioma-associated epilepsy treatment.

Additionally, STAT6 is an important target protein of IL-4 that likely regulates the microenvironment of tumor cell growth, inhibits tumor invasion, and reduces tumor proliferation and differentiation (Rahaman et al., 2005; Hammond et al., 2018). Both in human glioma tissue and glioblastoma cells (U87MG and U373MG), Park et al. (2019) found that CpG islands in the STAT6 promoter were hypermethylated by DNA methyltransferase which ultimately led to the down-regulated and even silenced expression of STAT6. Furthermore, under hypoxic conditions, the decrease of STAT6 could activate the mTOR signaling pathway, promote hypoxia-inducible factor-1 (HIF-1α) protein synthesis, and eventually enhance the viability and anti-apoptotic ability of tumor cells. Using DNA methyltransferase inhibitors, such as five-azacitidine and decitabine, to restore STAT6 expression in STAT6-silenced gliomas can increase tumor cell death, which would provide a new treatment (Park et al., 2019). Interestingly, in glioblastoma cells (U251, T98G, and A172), Rahaman et al. found that IL-4 induced the abnormal activation of STAT3 in GBM cells, but not in normal human astrocytes. These results indicate that IL-13Rα2, a decoy receptor for IL-13, negatively regulates STAT6 activation and positively regulates IL-4R/IL-13R-mediated STAT3 activation in GBM cells. Moreover, this work also demonstrated that the IL-13Rα2-mediated activation of STAT3 did not need a direct physical interaction between IL-13Rα2 and STAT3 (Rahaman et al., 2005). Thus, as IL-4 induces a similar response in intractable epilepsy and glioma, IL-4/IL4R-STAT6 may be a potential therapeutic target for glioma-related epilepsy but requires future studies.

## Il-4, Epilepsy and Cognitive Function

The temporal lobe is thought to influence cognition and memory, especially in the spatial domain. Chronic recurrent temporal lobe epilepsy can lead to dramatic cognitive impairment (Chauvière, 2020). Many reports have found that children with epilepsy have high levels of cognitive disorders, which is mainly influenced by the etiology, time of seizures, frequency of interictal epileptiform discharges, and the adverse reactions of antiepileptic drugs (AED) or surgery (Moosa and Wyllie, 2017). A recent registration study of 6,635 children with epilepsy in Norway showed that 17.0% have intellectual disabilities, 21.3% have mental developmental disabilities, and 7.5% have unexplained developmental delays (Aaberg et al., 2016). In children who have had seizures at least once in the past year or used AEDs, 40% had an IQ less than 70 and 24% under 50 (Reilly et al., 2015). Cognitive impairment imposes a serious burden on patients and families and therefore, early improvement of cognitive impairment will help improve the patients’ quality-of-life.

A recent study discovered that IL-4 is beneficial to cognition. In the meninges of Morris water maze (MWM)-trained mice, CD4+ T cells were activated and produced more IL-4 than untrained controls. IL-4^−/−^ mice exhibited severe cognitive impairment and the level of pro-inflammatory CKs was increased. Subsequently, the bone marrow of these IL-4^−/−^ mice was transplanted into wild-type mice, which also showed severe cognitive impairment and revealed a marked pro-inflammatory skew in meningeal myeloid cells. On the other hand, injecting wild-type mouse T cells into IL-4^−/−^ mice could significantly improve cognitive function and ameliorated the pro-inflammatory tendency of meningeal cells. Derecki et al. (2010) also found IL-4 increased brain-derived neurotrophic factor (BNDF) mRNA levels in astrocytes. Wild type mice that were tested in the MWM appeared to accumulate IL-4 and IL-13 in their meninges and BDNF in the hippocampus continued to rise. Overall, both IL-4 and IL-13 deficiency in the brains of these mice could damage spatial learning. Consequently, both IL-4 and IL-13 are involved in cognitive function by stimulating astrocytes from the meninges and hippocampus (Brombacher et al., 2017). Additionally, in the hippocampus of aged Sprague-Dawley male rats, the level of BDNF and synaptophysin is reduced, pro-inflammatory cytokine (IL-1β and IL-6) to be released, and cognitive dysfunction present. Treatment with IL-4 reversed BDNF and synaptophysin expression promoted the transformation of microglia to the M2 phenotype, downregulated the expression of IL-1β and IL-6, and improved the behavioral performance (Li et al., 2017).

BDNF and synaptophysin regulated by astrocytes are both important for cognitive processes. BDNF can regulate neuroplasticity, including long-term potentiation, synaptogenesis, and neurogenesis, all of which are related to learning and memory (Leal et al., 2017). Specifically, synaptophysin plays a crucial role in the regulation of synaptic plasticity that, when disordered, can result in the cognitive decline of AD (Valtorta et al., 2004). Nowadays, several studies have shown that IL-4 might have potential neuroprotective effects on cognition by regulating BDNF and synaptophysin. Nevertheless, this concept still requires further studies to show whether IL-4 can act as a cognitive protector in epilepsy-related cognitive impairment.

## Summary

**Figure 3:**
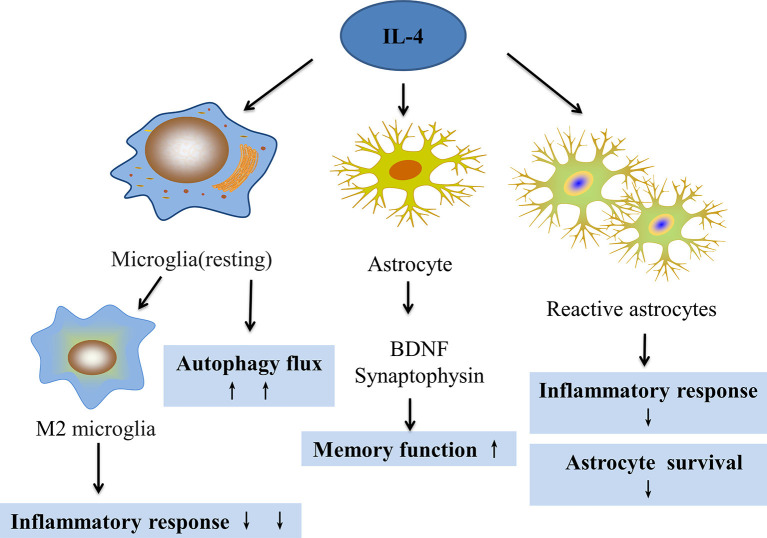
The effect of IL-4 on microglia and astrocytes. IL-4 can regulate microglial transformation into the M2 phenotype to reduce inflammation, increase autophagy flux, and improve autophagy. IL-4 can also increase the expression of BDNF and synaptophysin in astrocytes, which can improve memory. IL-4 can also reduce the proliferation of reactive astrocytes and reduce the inflammatory response.

Increasing evidence has revealed that inflammation and epilepsy exhibit complex interactions. Microglia and astrocytes are the main inflammatory cells in the nervous system that are involved in the pathogenesis of epilepsy. This pathogenesis includes activating M1 microglia, releasing pro-inflammatory factors, and stimulating the proliferation of reactive astrocytes that form glial scars and cause hippocampal sclerosis. IL-4 is an important anti-inflammatory cytokine that has an important regulatory effect on the above response processes of glial cells (Figure 3). Furthermore, IL-4/STAT6 might be an important pathway for regulating multiple aspects of the activated glial pathology. Additionally, IL-4 can affect the prognosis of gliomas, which are closely related to epilepsy, and improve cognitive function. Moreover, in the adult zebrafish brain, Bhattarai et al. (2016) found that IL-4 regulated neurogenesis, especially NSC proliferation through the STAT6 pathway, and also promoted neural stem cell proliferation and neurogenesis by inhibiting tryptophan metabolism. These events reduce serotonin production and up-regulate BDNF (Bhattarai et al., 2020). Whether in animal models with epilepsy or patients with temporal lobe epilepsy, neurogenesis was reported to be involved in epileptogenesis and epilepsy outcomes (Chen et al., 2019). Consequently, IL-4 very likely plays an important role in the development of epilepsy by regulating neuron-glial interactions.

## Author Contributions

LChen, LZ, DL, ZW, and PX contributed to the conception and design of the study. LChen to the writing of the article. QW and YH were responsible for funding. YH contributed to revising the article. LChang and QW were responsible for scientific consultation at all stages, the conceptualization of the study, and revision of the article.

## Conflict of Interest

The authors declare that the research was conducted in the absence of any commercial or financial relationships that could be construed as a potential conflict of interest.

## Abbreviations

CKs: Cytokines
IL-1β: interleukin-1β
IL-4: Interleukin 4
IL4Rα: interleukin 4 receptor alpha
γc: r chain
IL-13Rα1: interleukin 13 receptor α1
IFN-γ: Interferon γ
TNF-α: tumor necrosis factor-α
MHC II: major histocompatibility complex II
SE: status epilepticus
FS: febrile seizures
CORT: corticosterone
PGE2: prostaglandin E2
5-HT: serotonin
NE: norepinephrine
JAK: Janus kinase
IRS: Insulin receptor substrate protein
PI3K: Phosphoinositide-3 kinase
AKT: Protein kinase B
STAT6: Signal transducer and activator of transcription factor
mTOR: Mammalian target of rapamycin
P2Y1: Purinergic Receptor 1
EAE: experimental allergic encephalomyelitis
MOGp: myelin oligodendrocyte glycoprotein
MSCs: mesenchymal stem cells
CPZ: Cuprizone
KA: Kainic acid
EEG: electroencephalographic
LPS: lipopolysaccharide
MTLE-HS: mesial temporal lobe epilepsy with hippocampal sclerosis
ADP: adenosine diphosphate
PPARγ: peroxisome proliferator activated receptor γ
AD: Alzheimer’s disease
MTLE: mesial temporal lobe epilepsy
HS: hippocampal sclerosis
RE: Rasmussen’s encephalitis
HMGB-1: High mobility group box-1 protein
DAMP: Damage Associated Molecular Patterns
NF-κB: nuclear factor-kB
NLRP3: NOD-like receptor with pyrin domain containing-3
OLCs: oligodendroglia-like cells
GRE: glioma-associated epilepsy
LGG: low-grade gliomas
GBM: glioblastoma
HIF-1α: hypoxia-inducible factor-1
AED: antiepileptic drugs
MWM: Morris water maze
BNDF: brain-derived neurotrophic factor
SPR: surface plasmon resonance.
